# Patient-Derived Lung Cancer “Sandwich Cultures” with a Preserved Tumor Microenvironment

**DOI:** 10.1089/ten.tec.2023.0199

**Published:** 2024-01-17

**Authors:** Hailong Wang, Thorsten Walles, Cornelia Wiese-Rischke

**Affiliations:** University Clinic for Cardiac and Thoracic Surgery, Otto-von-Guericke-University Magdeburg, Magdeburg, Germany.

**Keywords:** NSCLC, 3D lung cancer model, TME, dynamic 3D model

## Abstract

In the past, different spheroid-, organotypic-, and three-dimensional (3D) bioprinting lung cancer models were established for *in vitro* drug testing and personalized medicine. These tissue models cannot depict the tumor microenvironment (TME) and, therefore, research addressing tumor cell–TME interactions is limited. To overcome this hurdle, we applied patient-derived lung tumor samples to establish new *in vitro* models. To analyze the tissue model properties, we established two-dimensional (2D) and 3D coculture tissue models exposed to static and dynamic culture conditions that afforded tissue culture for up to 28 days. Our tissue models were characterized by hematoxylin eosin staining, M30 enzyme-linked immunosorbent assay, and immunofluorescence staining against specific lung cancer markers (TTF-1 and p40/p63), cancer-associated fibroblast (CAF) markers (α-SMA and MCT4), and fibronectin (FN). The 3D models were generated with higher success rate than the corresponding 2D model. The cell density of the static 3D model increased from 21 to 28 days, whereas the apoptosis decreased. The dynamic 3D model possessed an even higher cell density than the static 3D model. We identified lung cancer cells, CAFs, and FN. Therefore, a novel *in vitro* 3D lung cancer model was established, which simulated the TME for 28 days and possessed a structural complexity.

**Impact statement:**

It is becoming increasingly clear that tumor structure and microenvironment (TME) of solid tumors have a relevant influence on the response to drug therapies. Both tissue properties are unfortunately insufficiently represented in the established *in vitro* models. In this study, we introduce a culture model that maintains TME and affords tissue culture for at least 4 weeks.

## Introduction

Lung cancer is the leading cause of cancer-related death worldwide, with a 5-year survival rate of only 26% for non-small-cell lung cancer (NSCLC).^1^ In lung cancer research, patient-derived three-dimensional (3D) *in vitro* tissue models are promising tools for translational research as well as personalized medicine.^2^ Different concepts have been introduced to emulate the NSCLC tumor niche *in vitro*, such as spheroids, or scaffold-based and microfluidic models.^3–6^ These models recapitulated the cytological features and markers of lung cancer, and demonstrated the utility of drug screening and separation of cancer cells from liquid biopsy. However, the existing 3D models lack the tumor microenvironment (TME), which consists of for example, cancer-associated fibroblasts (CAFs), immune cells, and the interaction with cancer cells.^7^ Multitudinous studies carved out the critical role of the TME in cancer development, progression, and metastasis.^8,9^ The absence of TME in experimental lung cancer research alters spatial distribution and gene expression of lung cancer cells and changes their response to drugs.^10^

Therefore, it is our intention to generate an *in vitro* 3D lung cancer model with a preserved TME.

In this study, we report our proof of concept and highlight the existing limitations of our new experimental research approach.

## Materials and Methods

### Patient-derived tumor samples

Tissue specimens from the tumor tissue were obtained from patients undergoing elective pulmonary resection for NSCLC at the University Hospital Magdeburg. Patients' informed consent was obtained before surgery. The study was approved by the ethics committee of the medical faculty of the Otto-von-Guericke-University Magdeburg (vote 163/17, on 16 October 2017). Tumors were sampled after surgical resection by a pathologist to guarantee clear resection margins, transported on ice, and stored in HBSS at 4°C until further processing. One half of the tumor tissue was embedded with Tissue-Tek^®^ O.C.T. Compound (Sakura, Umkirch, Germany), and stored at −80°C as positive control. The other half was minced into 1–1.5 mm^3^ pieces for two-dimensional (2D) and 3D culture experiments. All tumor pieces were washed three times with phosphate-buffered saline (PBS) to remove blood and tissue debris.

### 2D cell culture

Tumor pieces were digested either with 2.5 mg/mL Collagenase A (Roche, Mannheim, Germany) or 1 mg/mL Collagenase type IV (Sigma-Aldrich, Munich, Germany). The amount of enzyme solution corresponded to five times the volume of the tumor pieces. The samples were shaken continuously in the water bath at 37°C. Every 30 min, the supernatant was collected, and the digestion was stopped with the same volume of serum-containing medium, while the solid residues were incubated with fresh enzyme solution. After all solid tissue was digested, cells were centrifuged at 200 g for 3 min. Cell pellets were suspended with Dulbecco's modified Eagle's medium (DMEM) (Thermo Fisher Scientific, Waltham, MA), 10% standardized fetal bovine serum (FBS SUPERIOR stabil^®^) (Bio & Sell GmbH, Feucht, Germany), and 1% antibiotic–antimycotic solution (Sigma-Aldrich). The cells were cultured for 21 days on 6 cm Petri dishes under standard culture conditions in a humidified incubator containing 5% CO_2_ at 37°C. Cell culture media were exchanged every 2–3 days.

### Generation and cultivation of a static 3D lung cancer model

**FIG. 1.**
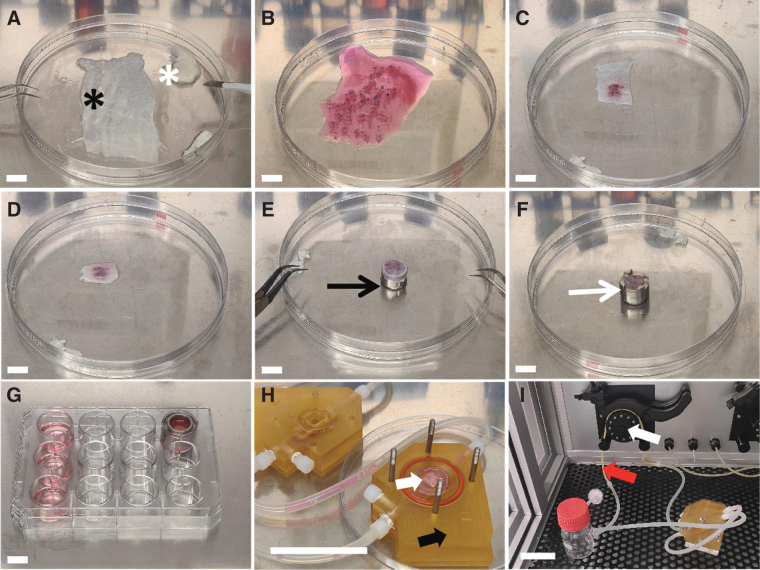
Generation and cultivation of the applied 3D tissue models. **(A)** The SIS biological matrix (*black asterisk*) is trimmed to fit the customized receptacle (cell crown). Residues are removed (*white asterisk*). **(B)** Tumor tissue form surgical specimen is cut into 1–1.5-mm^3^pieces. **(C, D)** Tumor pieces are embedded between two layers of trimmed SIS. **(E, F)** The “sandwich culture” is stretched on the inner metal crown (*black arrow*) and fixed with the outer metal crown (*white arrow*). **(G)** The “sandwich” model is covered with culture medium and cultured in a 12-well plate. **(H)** For dynamic tissue culture conditions, the “sandwich” model (*white arrow*) was transferred onto the bioreactor platform (*black arrow*). **(I)** A pulsatile medium flow is established using a closed perfusion circuit (*red arrow*) and a peristaltic roller pump (*white arrow*). Scale bar represents in **(A–G)** 1 cm and in **(H, I)** 6 cm. 3D, three-dimensional; SIS, small intestinal submucosa.

For static 3D cultivation, we developed a “sandwich” culture method: 10 tumor pieces were embedded between two layers of small intestinal submucosa (SIS). The SIS was prepared from decellularized porcine jejunum as described before (Fraunhofer Institute for Silicate Research ISC, Würzburg, Germany).^11,12^ The mucosa, the remaining vascular tree, and the serosa were removed and the scaffold was cut into 1.5 × 3 cm pieces (Fig. 1A–G). The tumor pieces were seeded between the two layers of the scaffold without air bubbles. The “sandwich” 3D model was fixed between two customized cylinders (cell crowns), transferred to a 12-well plate (Thermo Fisher Scientific), and cultured in DMEM with 10% FCS and 1% antibiotic–antimycotic solution. Tissue cultures were checked for contamination with the microscope (Evos XL Core AMEX 1000; Thermo Fisher, Bothell, WA, 20 × objective) after overnight cultivation. The medium was exchanged every 2–3 days.

The models were cultured for 21, 28, and 42 days, respectively, under standard conditions. At the respective days, they were embedded with Tissue-Tek O.C.T. Compound (Sakura) and stored at −80°C for further analysis.

### Generation and cultivation of a dynamic 3D lung cancer model

The 3D models were cultured overnight. The next day, they were transferred into the bioreactor chamber for dynamic culture (Fig. 1H, I). The customized bioreactors (described before^13^) were perfused with tissue culture medium (50 mL DMEM with 10% FCS and 1% antibiotic–antimycotic solution) using a peristaltic pump at a speed of 3 rpm providing a medium flow of 1.5 mL/min. The medium was exchanged every 7 days. The 3D models were cultured for 21 and 28 days, respectively, under standard conditions. At the end of the experiment, they were embedded and stored as described before.

### Cocultivation with either human bronchial fibroblasts or NIH-3T3 cell line

Human primary bronchial fibroblasts (hbFb) isolated from bronchus were cultivated with DMEM containing 10% FCS. The fibroblast cell line NIH-3T3 isolated from a mouse NIH/Swiss embryo was kindly provided by Prof. Dr. Andrea Kröger (Helmholtz-Center, Braunschweig, Germany) and was cultured in DMEM with 10% FCS. The NIH-3T3 cells were mitotically inactivated using 4 μg/mL mitomycin C (Sigma) for 2 h kept under standard culture conditions and afterward washed five times with PBS. For cocultivation, hbFbs or mitotically inactivated NIH-3T3 cells were seeded in a 12-well plate and cultivated under standard conditions. When the hbFbs reached a cell confluency of 80%, cell crowns of the static 3D models were transferred into the corresponding wells and cultured with DMEM with 10% FCS and 1% antibiotic–antimycotic solution. Wells containing hbFb or mitotically inactivated NIH-3T3 were replaced every week and every 2 weeks, respectively.

### Histological and immunofluorescence staining

Frozen samples were cut into 10 μm cryosections using a Leica CM1950 cryomicrotome (Leica, Deer Park). Hematoxylin–Eosin (HE; C. Roth GmbH, Karlsruhe, Germany) staining was done according to standard protocols. For immunofluorescence staining, samples were fixated using a 4% paraformaldehyde solution (C. Roth GmbH) for 10 min at room temperature (RT). Samples were blocked with either 3% goat or donkey serum in PBS for 30 min at RT. Primary antibodies were diluted in blocking serum and incubated overnight at 4°C in a humidified chamber. The following primary antibodies were used: mouse anti-p40 (1:50, clone BC28, Zytomed Systems, Berlin, Germany), rabbit anti-p63 (1:50; Gene Tex, CA), mouse anti-TTF-1 (1:100, clone 8G7G3/1; Zytomed Systems), mouse anti-α-SMA (1:100, clone 1A4; Invitrogen, Thermo Fisher Scientific), rabbit anti-MCT4 (1:100; Novus Biological, Abingdon, United Kingdom), and mouse anti-fibronectin (FN) (1:100, clone FBN11; Invitrogen).

Secondary antibodies were Cy3-conjugated donkey anti-mouse IgG (1:500; Jackson ImmunoResearch, Ely, Cambridgeshire, United Kingdom), Cy3-conjugated goat anti-rabbit IgG (H+L) (1:500; Jackson ImmunoResearch), and Cy3-conjugated goat anti-mouse IgG2a (1:500; Jackson ImmunoResearch). They were diluted in blocking serum and incubated for 1 h at RT. After removal of the antibody, sections were incubated with 1 μg/mL 4′,6-diamidino-2-phenylindole (DAPI) (nuclear stain; Sigma-Aldrich) in PBS for 10 min at RT. Sections were washed three times in PBS and embedded in Mowiol 4-88 with DABCO (2.5%) (Carl Roth GmbH, Hamburg, Germany). The immunofluorescence staining was repeated three times with negative control (omitting the primary antibody). HE staining images and immunofluorescence staining images were obtained using Invitrogen EVOS™ XL Core Imaging System and EVOS FL Auto 2.0 Imaging system (both from Thermo Fisher Scientific), respectively.

### M30 enzyme-linked immunosorbent assay (apoptosis)

To determine epithelial cell-derived apoptosis, we used the M30 CytoDeath™ enzyme-linked immunosorbent assay (ELISA) (TECOmedical AG, Sissach, Switzerland) for the quantitative determination of the soluble caspase-cleaved keratin 18 (cck18) Asp396 (M30) neo-epitope. The release of the antigen into the cultivation medium occurs due to secondary necrosis of apoptotic bodies. Sample media were collected every 7 days from each 3D model. The ELISA was performed according to the manufacturer's protocol. Samples were measured in duplicates: Samples with a too high concentration were diluted to half with the corresponding cell culture medium to fit the range of the standard curve from 0 to 3000 U/L. After incubation on an orbital shaker (600 rpm) for 4 h, wells were washed five times before the addition of the substrate. The reaction was stopped after 20 min. Absorbance was measured with a microplate reader (TECAN, Männedorf, Switzerland) after 10 s shaking and 5 min of waiting. The concentrations of cck18 were calculated according to the standard curve fitted with cubic spline algorithm.

### Success rate evaluation

We applied the pathological classification of clinical lung cancer samples to match our models.^14,15^ In the 2D culture, successful cell cultivation was determined by morphological appearance using light microscopy according to the following criteria: (1) presence of two or more different cell types, (2) lung cancer cell colonies showing irregular cell size and shape; enlarged nuclei; increased nuclei–cytoplasm ratio; abnormal cellular structures, such as cytoplasmic vacuoles and multinuclei or intercellular bridges. In 3D culture, criteria were evaluated following HE and immunofluorescence staining: (1) the presence of two or more different cell types, (2) the presence of two or more tumor-specific structures (cancer colony; tissue necrosis; cell invasion into normal tissue), and (3) expression of lung cancer-specific markers (TTF-1, p40/p63) according to the pathology report of the patients.

### Thickness and cell density evaluation of 3D model

The thickness of 3D tissue models was measured using the software ImageJ 1.52d (Wayne Rasband; National Institutes of Health). Five tiled images with 10-fold magnification were selected from nonoverlapping regions of each sample. The images should contain cells identified by DAPI staining, original tumor pieces, and the biological matrix identified by autofluorescence. With the threshold, the selection area and segmented line tool in ImageJ, the area, and the centerline of each 3D model were measured. The thickness was calculated by the following formula:

$$Thickness\left(\mu m\right)=\frac{Areaof3Dmodel\left(\mu m^{2}\right)}{Centerline\left(\mu m\right)}$$

Cells in the 3D model were counted using the same software ImageJ automatically and by manual counting. Five nonoverlapping images (10-fold magnification) and five overview images (tiled with 10-fold magnification) of DAPI staining per model were used to determine the cell number through ImageJ Macro (Supplementary Data S1). The merged DAPI staining and autofluorescence images were used to measure the area of the 3D model. In the 10-fold magnification image, two layers of matrix, tumor pieces, and cells should be included. The cell density was calculated by the following formula:

$$Celldensity\left(cells∕mm^{2}\right)=\frac{Cellnumber\left(cells\right)}{Areaof3Dmodel\left(mm^{2}\right)}$$

### Statistical analyses

Statistical significance of the success rate and the lung cancer colony rate was determined using the Fisher's exact test. The unpaired *t*-tests or the one-way analysis of variance (ANOVA) and Tukey's test were used for analyzing the thickness and the cell density in static 3D model and the cell density comparison of the static 3D model and the dynamic 3D model. Mann–Whitney U test was used to analyze the thickness differences between static and dynamic 3D models. Kruskal–Wallis test was used for comparing multiple groups (M30 ELISA). The *p*-values ≤0.05 were considered significant; *p ≤ 0.05; **p ≤ 0.01; ***p ≤ 0.001. Diagrams were designed with Prism 9 (GraphPad, San Diego).

## Experiment

### General information about the patients

In this study, tumor samples from 21 patients (age range 48–83 years) were analyzed in 2D and 3D culture. According to clinical pathology reports, 1 case was a small cell lung cancer (SCLC) and 20 cases were NSCLC with 14 cases of adenocarcinoma (ADC) and 6 cases of squamous cell carcinoma (SQCC). Patient information is included in the Supplementary Table S1.

### Success rate and lung cancer colony rate in 2D and static 3D culture

**FIG. 2.**
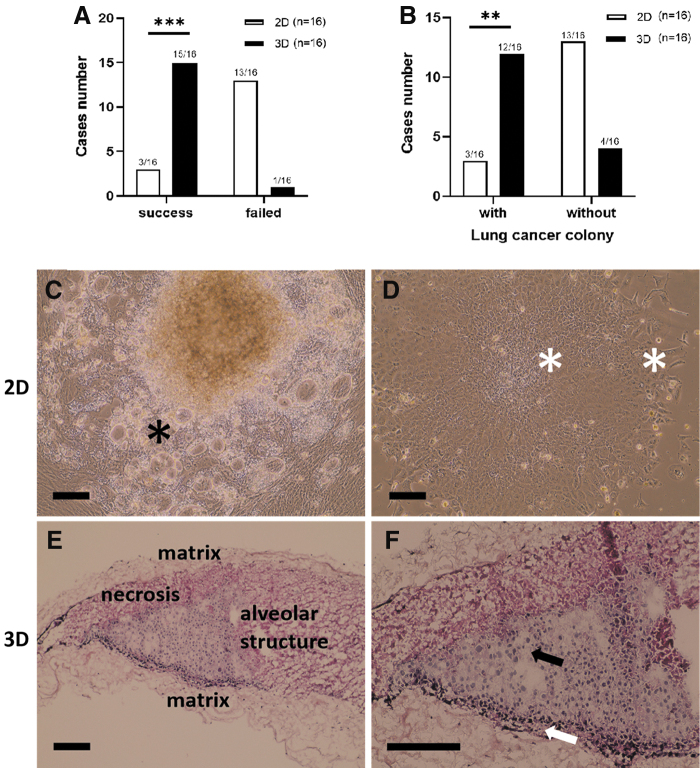
Comparison of 2D and static 3D models. **(A)** Success rate and **(B)** lung cancer colony rate in 2D and static 3D models. **(C–F)** Morphological features of 2D and static 3D models. **(C)** Lung cancer cells were identified by irregular cell size and shape, enlarged nucleus, and cytoplasmic vacuoles (*black asterisk*). **(D)** Increased nucleus–cytoplasm ratio, multinuclei, and intercellular bridges were identified in 2D culture (see *white asterisks*). **(E)** In HE stained sections of static 3D culture, there are two layers of matrix covering the original lung tumor pieces. Within the static 3D model, we could identify original alveolus structures and necrosis area. **(F)** Several types of cells existed in static 3D model with different morphology. Lung cancer cells are labeled with a *black arrow* and noncancer cells with a *white arrow*. Scale bar represents 200 μm. Significance was determined using the Fisher's exact test. *p ≤ 0.05; **p ≤ 0.01; ***p ≤ 0.001. 2D, two-dimensional; HE, hematoxylin–eosin.

First, we compared the success rate and the lung cancer colony rate of 2D and static 3D cultivation using the basal medium (DMEM with 10% FCS and antibiotics). According to the cell morphology, cultures with tumor cells were obtained from three samples in 2D and 15 samples in 3D after 28 days of cultivation (Fig. 2A). The success rate in 2D culture was 19% (3/16) with one ADC, two SQCC, and zero SCLC. In static 3D culture, the success rate was with 94% (15/16) (9 ADC, 5 SQCC, and 1 SCLC) significantly higher (*p* < 0.001). Lung cancer colonies were detected in three cases of 2D culture and in 12 cases of static 3D culture (Fig. 2B). The rate of lung cancer colony in the static 3D culture was with 75% (12/16) significantly higher than in 2D culture with 19% (3/16) (*p* < 0.01). These data suggest that static 3D culture effectively supported the *in vitro* cultivation of the tumor cells.

### Morphological features of the 2D and the static 3D culture model

In 2D culture, cells possessed a flat morphology and grew as monolayer. The cancer colonies were surrounded by fibroblast-like cells and possessed vacuoles in some of the cells (Fig. 2C) and were easy to identify at the beginning of cell culture. They possessed clear intercellular bridges and higher nucleus/cytoplasm ratio compared with other cells (Fig. 2D). The three-layer structure of the “sandwich” culture can be identified in the static 3D model (Fig. 2E, F). According to cell distribution in the static 3D model, we defined three regions: (1) tumor area, (2) interface between tumor and matrix, and (3) the matrix which was infiltrated with cells. The static 3D models exhibited cancer type-specific morphologies (Supplementary Fig. S1).

### Thickness and cell density of the static 3D model

**FIG. 3.**
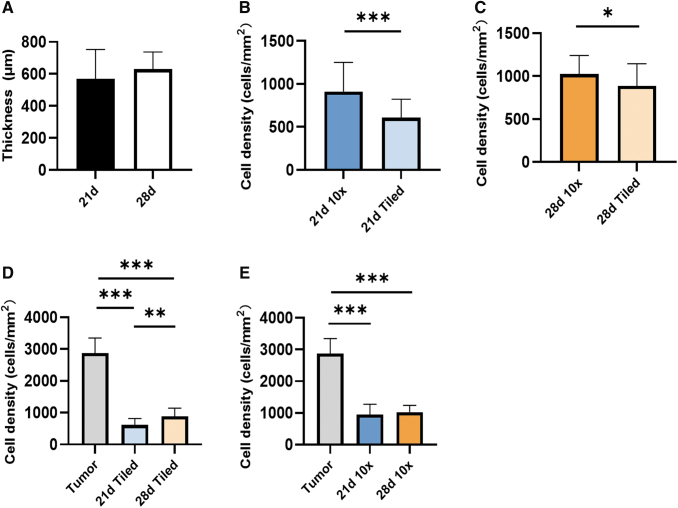
Thickness and cell density of the static 3D models. **(A)** Tissue thickness at days 21 and 28. **(B, C)** Cell density at day 21 **(B)** and day 28 **(C)** determined by 10-fold magnification (10 × ) and tiled images, respectively. **(D)** Comparison of the cell density at 21 and 28 days using tiled images (whole section) and **(E)** 10-fold magnification (10 × ) images (tumor area) with the original tumor. Significance was determined with the unpaired *t*-tests in **(A–C)** and the one-way ANOVA and Tukey's test in **(D, E)**. Shown are the mean with SD. *n* = 8, *p ≤ 0.05; **p ≤ 0.01; ***p ≤ 0.001. ANOVA, analysis of variance; SD, standard deviation.

To analyze cell growth in the static 3D model, we determined the tissue thickness and cell density. We utilized images focusing on the tumor area (10-fold magnification) and tiled images representing the whole section of the 3D static model. In the static 3D model, there was no significant difference in the thickness at days 21 and 28 (570 ± 183 μm vs 630 ± 108 μm, *p* = 0.0768) (Fig. 3A). In the tumor area (10-fold magnification), there was a higher cell density compared with the whole static 3D model (tiled images) irrespective of the analyzed time points (21 days, *p* < 0.001 or 28 days, *p* = 0.0116) (Fig. 3B, C). However, from day 21 to 28 cell density increased from 613 ± 204 cells/mm^2^ to 887 ± 259 cells/mm^2^ (*p* = 0.001) in the entire static 3D model (Fig. 3D), while there was no significant cell density increase in the tumor area (*p* = 0.58) (Fig. 3E). This suggests that during the fourth week of cultivation, the tumor cells proliferated mainly outside of the tumor piece area.

However, the cultivation time of 21 and 28 days, respectively, was still not sufficient for the static 3D models to develop the same cell density as determined in the original tumor with 2873 ± 473 cells/mm^2^ (*p* < 0.001).

### The TME of the static 3D model

To confirm the presence of lung cancer cells, we performed immunofluorescence stainings against the specific lung cancer markers according to the patient's clinical pathology reports. The tissue sections of the original tumor biopsies and the corresponding 3D model were stained in parallel. Comparing the data of the pathology report (Supplementary Table S1, see TTF-1/p63/p40 expression) with the staining results of the original tumor biopsies, in 11 out of 14 tumor biopsies, the marker expression was consistent with the pathology report. The tumor pieces within the 3D models showed strong autofluorescence and cancer cells grew in and around the tumor pieces. In 10 different 3D models, we confirmed the presence/absence of the lung cancer marker analyzed in the 19 corresponding original tumor biopsies (Supplementary Table S2).

**FIG. 4.**
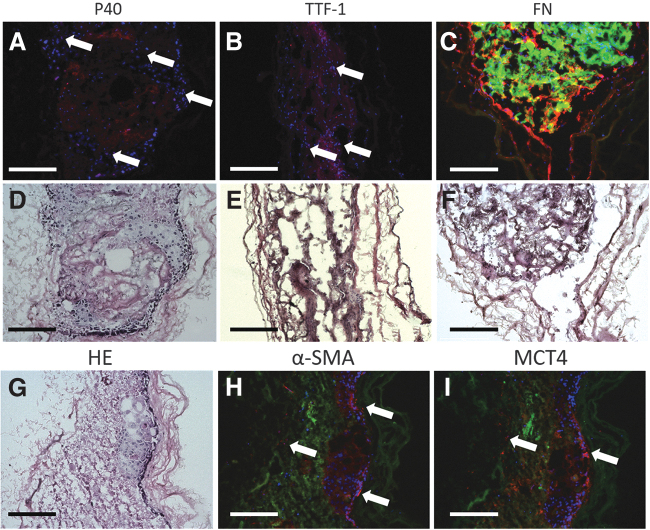
Immunostaining of the static 3D model. **(A, B)** Immunofluorescence staining against p40 and TTF-1 (*white arrows*). **(C)** Immunofluorescence staining against FN (*orange*). **(D–G)** The corresponding HE stainings showing the structures of the 3D models and morphology characteristics of the cells. **(H, I)** Immunofluorescence staining against α-SMA and MCT4 (*white arrows*). Autofluorescence of the tumor and matrix accounts for the orange fluorescence in **(A, B)** and green fluorescence in **(C, H, I)**. The nuclei were stained with DAPI (*blue*) in **(A–C)** and **(H–I)**. The scale bar represents 200 μm. DAPI, 4′,6-diamidino-2-phenylindole; FN, fibronectin.

In tumor marker-positive 3D models, we detected single cells positive for TTF-1 or p40/p63 (Fig. 4A, B) in the cancer colony (Fig. 4D, E). Additionally, we verified the presence of FN, a component of the extracellular matrix (ECM), and CAFs, which are both part of the TME. Strong FN signals were detected at the interface of tumor piece and matrix.

The FN filled the space between the tumor pieces and the matrix, extending along the latter (Fig. 4C, F). CAFs in our 3D model were identified by upregulation of α-SMA and MCT4. With the presence of α-SMA and MCT4, we proved that most CAFs assembled along the interface of tumor and matrix. Some CAFs grew within the matrix (Fig. 4G, H, I). In summary, these findings suggest that lung cancer cells, CAFs, and the ECM component FN formed a TME in the static 3D model.

### Static 3D models with cocultivation

**FIG. 5.**
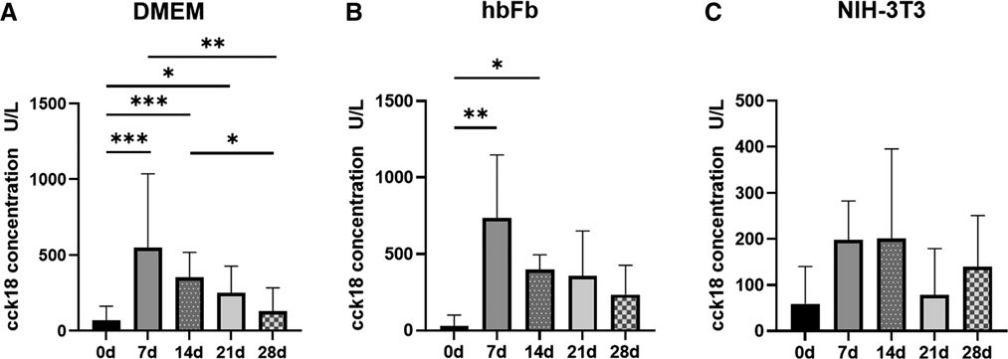
Measurement of apoptosis in the 3D model. The cck18 concentration in the medium of static 3D culture with DMEM; *n* = 10 **(A)**, in the medium of hbFb cocultures; *n* = 3 **(B)**, and in the medium of NIH-3T3 cocultures; *n* = 4 **(C)**. Statistical significance was determined using the Kruskal–Wallis test and Dunn's multiple comparisons test. Shown are the mean with SD. *p ≤ 0.05; **p ≤ 0.01; ***p ≤ 0.001. DMEM, Dulbecco's modified Eagle's medium; hbFb, human primary bronchial fibroblasts.

To support the growth of tumor cells in the 3D model, we tested two cocultivation approaches with different types of fibroblasts. Fibroblasts are known to produce and to secrete growth factors and cytokines that support tumor cell growth. Therefore, we used (1) hbFb and (2) mitotically inactivated NIH-3T3 cells. The latter have been already shown to support epithelial tumor cell growth *in vitro*.^16^ Suboptimal culture conditions have been shown to result in increased tumor cell apoptosis in *in vitro* culture models. Therefore, we first determined epithelial cell-specific apoptosis in the medium of the static 3D model cultivated with the DMEM basal medium. We observed that the concentration of cck18 was significantly higher at 7 and 14 days of cultivation compared with 28 days (Fig. 5A). The cck18 concentration at 7 days was above 500 U/L (549.74 ± 485.63) and decreased to 129.99 ± 153.94 U/L at 28 days. There was no significant difference of the cck18 concentration comparing 28 days and day 0 (*p* > 0.05). This may implicate that after 28 days of culture a homeostasis was achieved resulting in almost no apoptosis.

The quantification of apoptosis in the medium of the hbFb coculture showed a similar time course compared with the cultures with the DMEM. At day 7, we detected the highest level of cck18 that decreased with time to 234.67 ± 192.27 U/L at 28 days. However, we measured higher cck18 concentrations compared with the DMEM cultured models. This suggested that the coculture with hbFb was not as efficient as expected. In the second coculture experiments, we utilized mitotically inactivated NIH-3T3 cells. In this study, the overall level of cck18 was below 200 U/L. The highest concentration was measured at days 7 and 14, which decreased to 159.49 ± 104.30 U/L at 28 days and which was not significantly different from the negative control (day 0). These last data suggested that cocultivation with mitotically inactivated NIH-3T3 cells could support the tumor cell growth in the 3D model by reducing their apoptosis.

### Comparison of the static 3D model with the dynamic 3D model

**FIG. 6.**
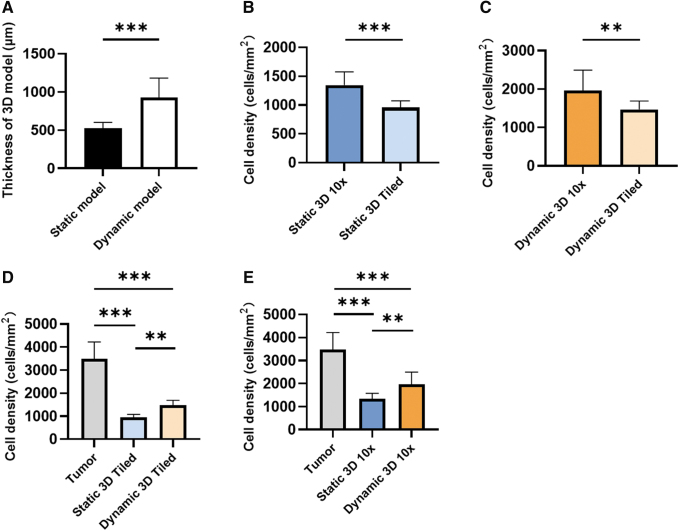
Comparison of the static and the dynamic 3D model. **(A)** Thickness of the static and the dynamic 3D model at 28 days. **(B)** Cell density of the static 3D model in 10-fold magnification (tumor area) and tiled images (whole section). **(C)** Cell density of the dynamic 3D model in 10-fold magnification and tiled images. **(D)** Comparison between static and dynamic 3D models with tiled image. **(E)** The comparison between static and dynamic 3D models with 10-fold magnification image. Statistical significance was determined using the Mann–Whitney *U* tests in **(A)**, unpaired *t*-test in **(B, C)**, and one-way ANOVA and Tukey's test in **(D, E)**. Shown are the mean with SD. *n* = 3, *p ≤ 0.05; **p ≤ 0.01; ***p ≤ 0.001.

In addition to the cocultivation approach, we tested whether continuous flow of the culture medium supports cell growth in the 3D model. We applied a constant medium flow of 1.5 mL/min that has been shown earlier to be suitable for the bioreactor used. Both static 3D models and dynamic 3D models of the same tumor biopsy were cultivated 28 days with the DMEM basal medium, and the thickness and cell density were measured to analyze the growth of the 3D model. The dynamic 3D models were thicker than the static 3D model (925 ± 256 μm vs 522 ± 78 μm, *p* < 0.001), suggesting more favorable growth conditions in the dynamic cultivation system (Fig. 6A). In coherence with our previous findings, tumor pieces had a higher cell density both in the static 3D model (*p* < 0.001, Fig. 6B) and the dynamic 3D model (*p* = 0.0026, Fig. 6C). In the tiled images, the cell density of the dynamic 3D model was higher compared with the static 3D model with 1470 ± 220 cells/mm^2^ and 956 ± 118 cells/mm^2^, respectively (*p* = 0.0086, Fig. 6D).

The 10-fold magnification images confirmed that the cell density of dynamic 3D model was higher than in the static 3D model (1961 ± 533 vs. 1345 ± 231 cells/mm^2^, *p* = 0.0091) (Fig. 6E).

**FIG. 7.**
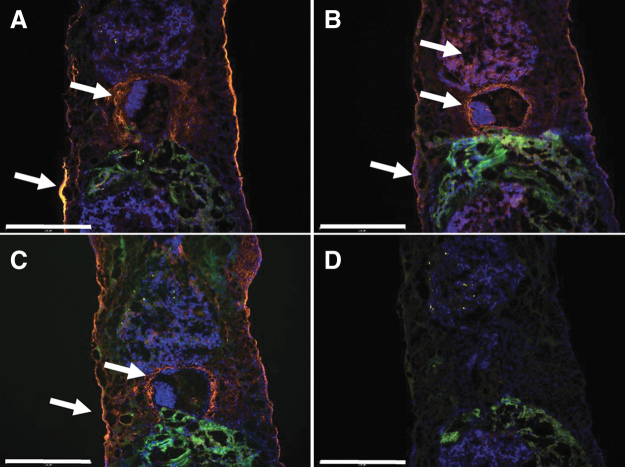
Immunostaining of the dynamic 3D model. **(A)** Immunofluorescence staining against α-SMA (*orange*). **(B)** Immunofluorescence staining against MCT4 (*orange*). **(C)** Immunofluorescence staining against FN (*orange*). **(D)** Negative control omitting the primary antibodies. The nuclei were stained with DAPI (*blue*) and the autofluorescence is seen by the strong green fluorescence. *White arrows* point to the fluorescence signals of the markers. Scale bars represent 500 μm.

These data suggest that the dynamic 3D models grew faster than the corresponding static 3D model. Although cell densities of the 3D models were lower than in the original tumor (3487 ± 736 cells/mm^2^), the dynamic 3D model showed higher cell density than the static 3D model, especially in the tumor pieces area. Next, we looked for the presence of CAF markers (α-SMA and MCT4) and FN in the dynamic 3D model (Fig. 7). Similar to the static 3D model, we found α-SMA- and MCT4-positive cells around the tumor pieces after 28 days of dynamic culture (Fig. 7A, B). FN was mainly present in the ECM surrounding the tumor pieces. These data suggested that the dynamic cultivation of the 3D sandwich model also supported the maintenance of the TME.

## Discussion

Organoids from bladder, breast, and metastatic colon cancer have been suggested as potential *in vitro* models for drug testing and personalized medicine.^17–19^ In contrast, organoids from NSCLC have a low establishment rate of only 17%.^20^ These *in vitro* models lack the original TME that is known to influence immune reaction and therapy.^21,22^ To circumvent these limitations, we used primary lung tumor pieces and a biological collagen matrix to establish a new *in vitro* 3D lung tumor model. This allowed us to maintain not only the tumor cells but also the spatial structure of the TME. After 28 days of cultivation, our 3D model showed a higher success rate and a higher cancer colony rate than the corresponding 2D model.

The tumor cell morphology and the tumor markers play an important role in the diagnosis of the lung cancer.^14^ Therefore, we utilized histological and immunostainings for the determination and characterization of the tumor cells in the 3D model. For immunostainings, we used the most common lung cancer markers TTF-1 for ADC and p40/p63 for SQCC according to the corresponding pathology report. In the 3D model compared with the original tumor biopsy, we confirmed the absence/presence of the tumor marker for 10 out of 19 cases. But the immunostainings demonstrated strong heterogeneity, because only few single cells were positive for the tumor markers. This could be due to the downregulation of marker expression during *in vitro* cultivation. Another reason could be a selection for specific cell types or outgrowth of nonmalignant cells.

In the 3D model, we found tumor pieces, which maintained a part of the original tumor morphology. ADC possessed flat, cohesive sheets of rather uniform-appearing glandular cells and the SQCC exhibited intercellular bridges as described earlier.^14^

We proved that the static 3D model possessed a TME, including CAFs and FN. Fibroblasts positive for α-SMA and MCT4 are considered to be CAFs.^23,24^ Catabolic CAFs (MCT4-positive) are known to play an important role in enabling cancer cell propagation, survival, and systemic dissemination during metastasis.^25^ FN is primarily synthesized by CAFs and deposits into the ECM that acts as scaffold for molecules such as growth factors and cell surface receptors.^26^ Due to the same distribution of FN and CAFs on the interface of cancer colony and matrix, we believe that the linear FN was produced by the CAFs. In the stroma of NSCLC, FN was overexpressed and promoted cancer cell adhesion, growth, differentiation, migration, invasion, survival, and resistance to chemotherapy.^27^ With the presence of CAFs and FN, therefore, our 3D model could be used for drug testing targeting CAFs and adjacent stroma.

The culture medium may also play an important role for cell growth and death. In the static 3D model with DMEM and with hbFb coculture, the apoptosis during the first 2 weeks was high, which then decreased to amounts lower than 200 U/L. However, coculture with mitotically inactivated NIH-3T3 cells resulted in constantly low apoptosis, which may support the growth of the tumor cells. The supplementation with the Rho kinase (ROCK) inhibitor compound, Y-2763, has been shown to minimize also cell death and increase organoid outgrowth.^28^ In future experiments, we plan to test the influence of the ROCK inhibitor.

To improve medium supply and metabolic waste transfer, we established the dynamic 3D model. Compared with the static 3D model, the dynamic 3D model was thicker and possessed higher cell density similar to the dynamic cultivation of human mesenchymal stem cells.^29^ The tumor pieces' area still showed the highest cell density. With sufficient medium supporting and growth space, our 3D model has the potential for long-term culture (more than 3 months).^30^

## Conclusion

We established a new 3D *in vitro* lung cancer model simulating the TME for 28 days. The 3D model showed a higher success rate and structural complexity than 2D models, which makes them suitable for future translational and oncological research.

## Supplementary Material

Supplemental data
