# Supplementary material for: Patient-Derived Lung Cancer “Sandwich Cultures” with a Preserved Tumor Microenvironment
Source: Tissue Eng Part C Methods. 2024 Jan 17;30(1):27–37. doi: 10.1089/ten.tec.2023.0199 (PMC10818046; doi:10.1089/ten.tec.2023.0199)
Supplement: Supplemental data [file Suppl_Data.pdf]

Squamous cell lung cancer

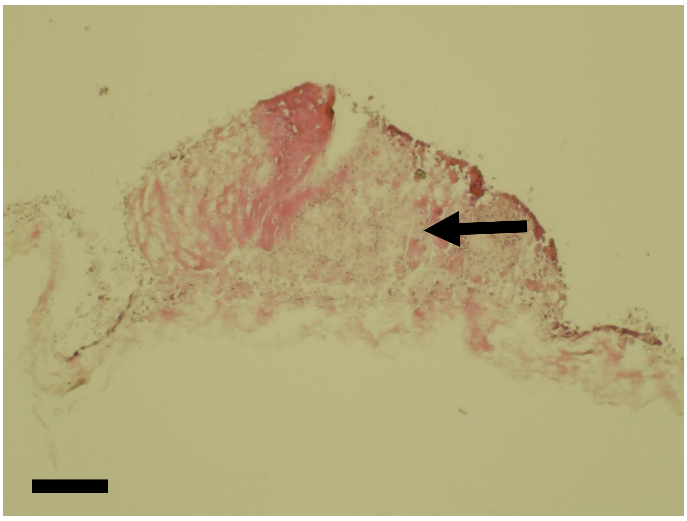

No.2 SQCC

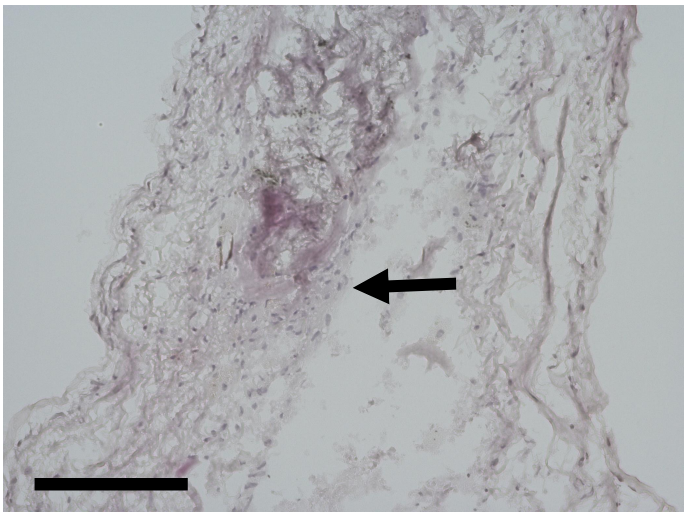

No.6 SQCC

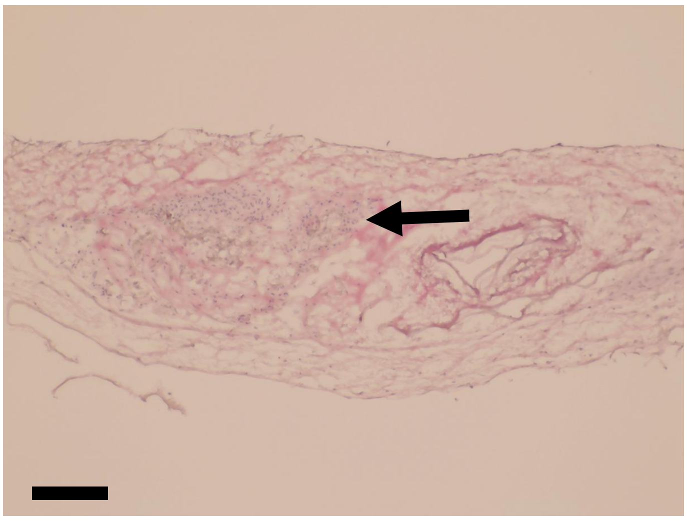

No.7 SQCC

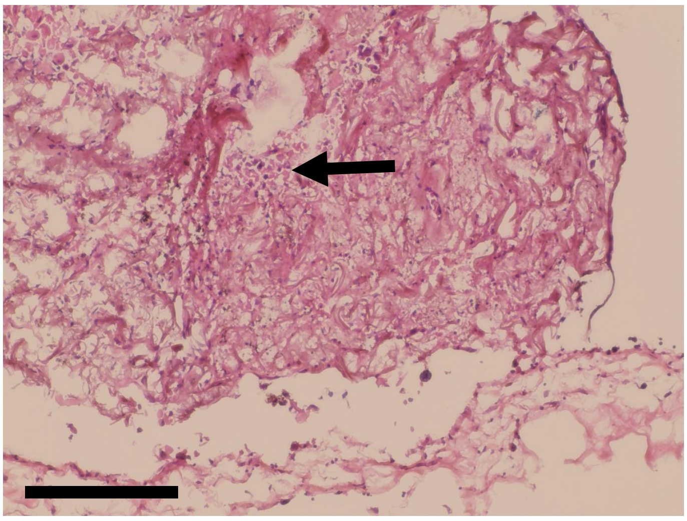

No.8 SQCC

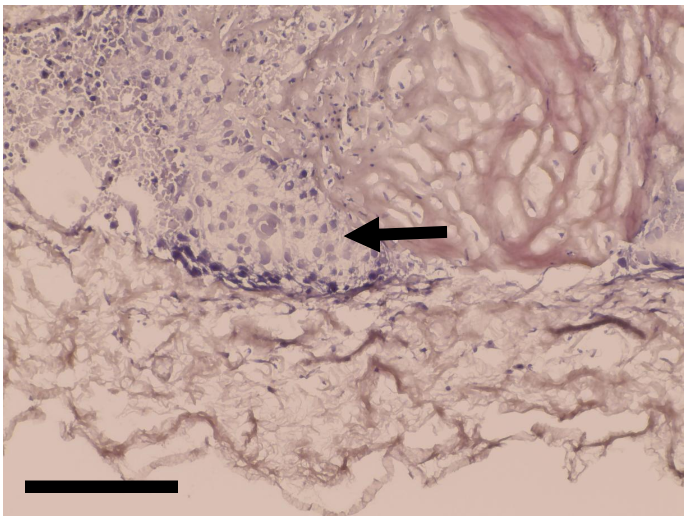

No.11 SQCC

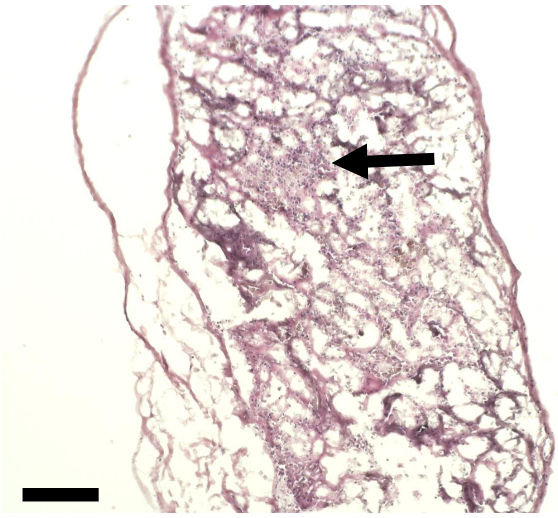

No.21 SQCC

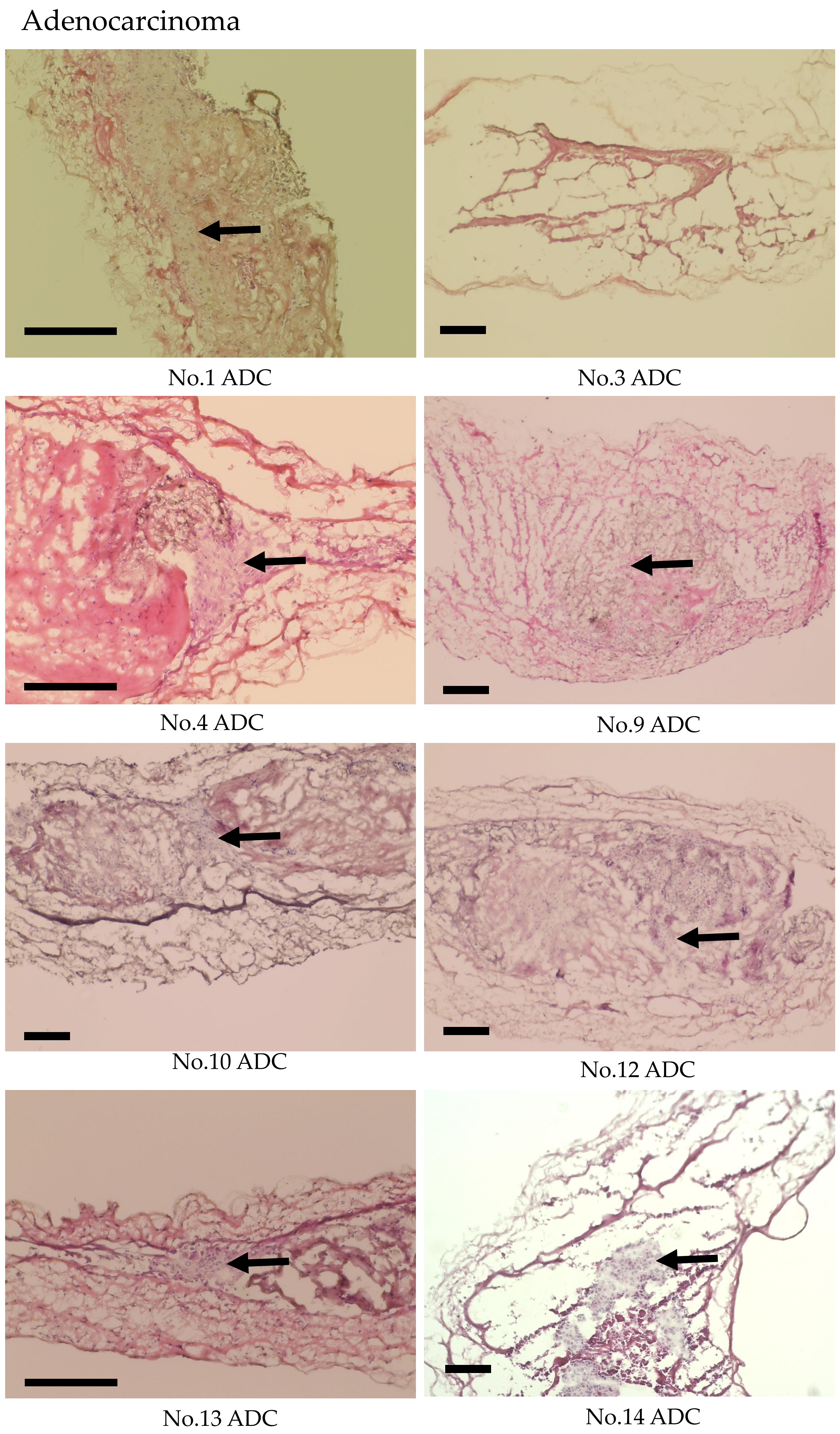
Adenocarcinoma
No.1 ADC
No.3 ADC
No.4 ADC
No.9 ADC
No.10 ADC
No.12 ADC
No.13 ADC
No.14 ADC

Adenocarcinoma

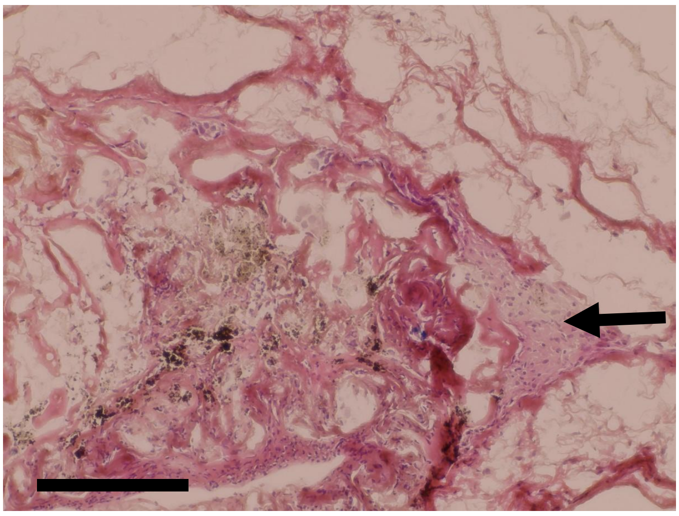

No.15 ADC

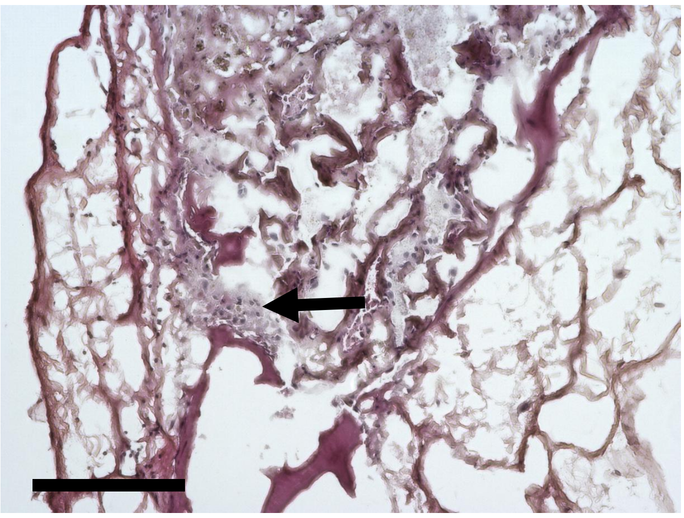

No.16 ADC

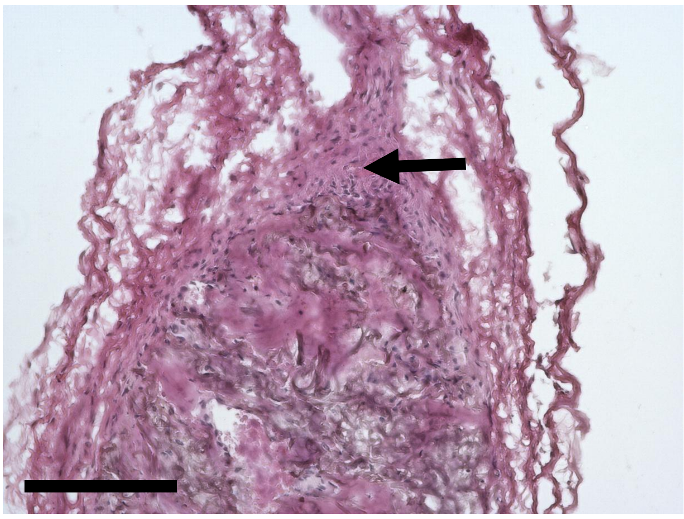

No.17 ADC

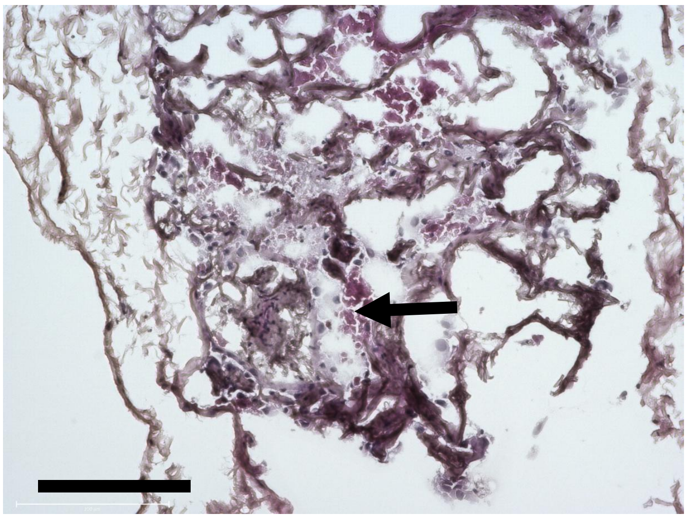

No.18 ADC

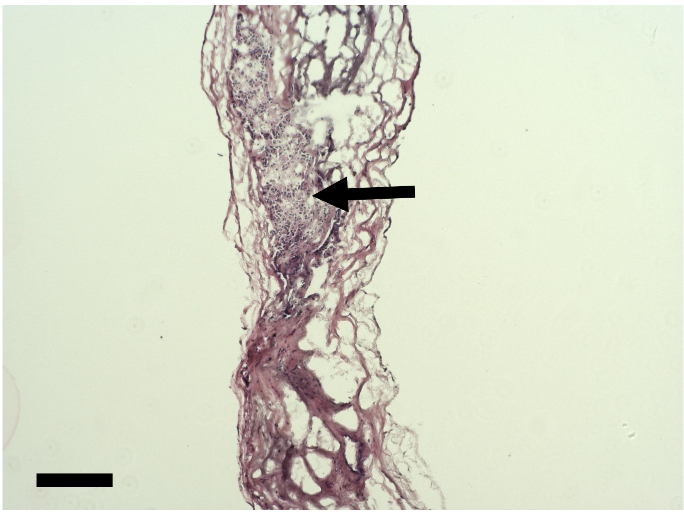

No.19 ADC

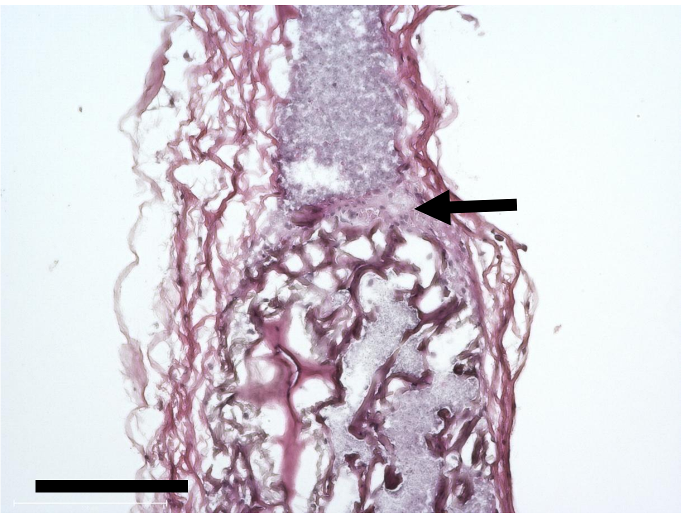

No.20 ADC

Small cell lung cancer

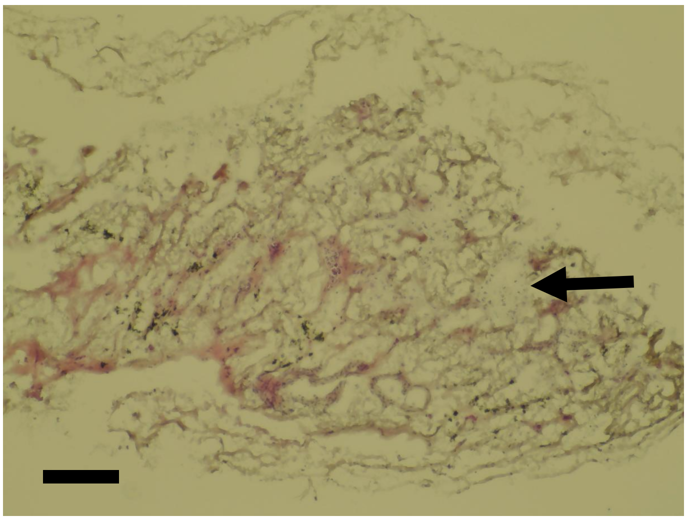

No.5 SCLC 10x

The Image J Macro used for counting DAPI-positive cells number.

```
run("8-bit");

//run("Brightness/Contrast...");

run("Enhance Contrast", "saturated=0.35");

setAutoThreshold("Default");

//run("Threshold...");

setAutoThreshold("Default dark");

//setThreshold(25, 255);

setOption("BlackBackground", false);

run("Convert to Mask");

run("Watershed");

run("Analyze Particles...", "size=10-Infinity show=Outlines display summarize in_situ");
```

Supplemental Figure legend:

Supplement Figure 1. The morphology of 3D models: SQCC: squamous cell carcinoma; SCLC: small cell lung cancer; ADC: adenocarcinoma. Black arrow: lung cancer cells. Scale bar represents 200 μm.

Wang H *et al.*
Patient-derived lung cancer “sandwich cultures” with a preserved tumor microenvironment

Supplement Table 1
Patient characteristics

Nr.	Patient number	gender	age (y)	BMI	FEV1 (%)	smoking history	pack years	histology	TTF-1/p63/p40 expression	TNM classification	tumor localization	induction therapy	type of surgery
1	#75	Male	61Y	18	77	smoker	30	pulmonary adenocarcinoma	Not mentioned	pT1b pN0(0/32) L0 V0 Pn0 R0 G3 cM0	left upper lobe	no	lobectomy
2	#76	Male	63Y	28	67	former smoker	55	pulmonary squamous cell carcinoma	p40+	pT3 pN0(0/31) L1 V0 Pn0 R0 G3 cM0	right upper lobe	no	lobectomy
3	#78	Male	78Y	34	56	former smoker	30 - 40	pulmonary adenocarcinoma	negativ	pT1c L1 V0 Pn0 R0 G2 cMx	right lower lobe	no	lobectomy
4	#79	Male	63Y	32	77	former smoker	30	pulmonary adenocarcinoma	TTF-1+	pT1c pN0(0/14) L0 V0 Pn0 R0 G2 cM0	right lower lobe	no	lobectomy
5	#83	Female	54Y	17	71	smoker	35	small cell lung cancer	negativ	ypT0 pN0 (0/7) V0 R0	right upper lobe	yes (chemotherapy with Carboplatin and Etoposid, mediastinale Rx with 66/2 Gy)	lobectomy
6	#84	Male	68Y	24	42	former smoker	30	pulmonary squamous cell carcinoma	p40+	pT4 pN1(1/17) L1 V1 Pn1 R0	left upper and lower lobe	no	pneumonectomy
7	#87	Male	65Y	25	-	former smoker	30	pulmonary squamous cell carcinom	Not mentioned	pT1c pN0 (0/18) L1 V0 Pn0 R0 G3 cM0	right lower lobe	no	segmentectomy
8	#89	Male	72Y	24	49	smoker	6	pulmonary squamous cell carcinom	p40+	pT2a pN0 L0 V1 R0 G2 cM0	right upper lobe	no	lobectomy
9	#91	Female	76Y	25	96	smoker	40	pulmonary adenocarcinoma	TTF-1+	pT2b pN0 (0/16) L0 V1 Pn0 R0 G3	left upper lobe	no	lobectomy
10	#92	Male	67Y	31	92	former smoker	20	pulmonary adenocarcinoma	TTF-1+	pT3 pN0 (0/4) L1 V1 Pn1 R0 G3 cM0	right lower lobe	no	bi-lobectomy
11	#94	Male	65Y	45	32	smoker	20	pulmonary squamous cell carcinom	p40+	pT4 pN0 (0/9) G3 L0 V0 R1	right lower lobe	no	lobectomy +segmentectomy
12	#96	Male	72Y	21	68	never smoker		pulmonary adenocarcinoma	TTF-1+	pT3 pN0 (0/17) L1 V1 Pn0 R0 G2 cM1b	left upper lobe	no	lobectomy
13	#107	Female	62Y	25	74	smoker	40	pulmonary adenocarcinoma	TTF-1+	pT2 pN1(1/10) L1 V1 Pn0 R0 G2	right upper lobe	no	lobectomy
14	#109	Male	51Y	29	78	smoker	30	pulmonary adenocarcinoma	TTF-1+	pT3 pN0 (0/35) G3 L0 V0 Pn0 R0 cM0	right upper lobe	no	lobectomy
15	#110	Female	48Y	35	79	smoker	20 - 30	pulmonary adenocarcinoma	TTF-1+	pT2a pN0 (0/22) L1 V0 Pn0 R0 G3 cM0	right upper lobe	no	lobectomy
16	#111	Female	61Y	24	61	smoker	50	pulmonary adenocarcinoma	TTF-1+	pT1c pN1 (1/5) L1 V0 Pn0 R0 G2 cM0	left upper lobe	no	lobectomy
17	#125	Female	83Y	20	108	former smoker	10	pulmonary adenocarcinoma	Not mentioned	pT1c pN0(0/22) L0 V1 Pn0 R0 G2	left upper lobe	no	lobectomy
18	#126	Female	78Y	20	-	never smoker		pulmonary adenocarcinoma	TTF-1 +	pT2a pN2(4/18) L1 V1 Pn0 R0 G2	left upper lobe	no	tri-segmentectomy
19	#130	Female	80Y	25	102	never smoker		pulmonary adenocarcinoma	TTF-1 weak positive	pT1b pN0(0/16) L1 V1 Pn0 R0 cM0	left lower lobe	no	lobectomy
20	#132	Female	65Y	19	90	never smoker		pulmonary adenocarcinoma	Not mentioned	pT4 pN0(0/40) L1 V0 Pn0 R0	right upper lobe	no	pneumonectomy
21	#135	Male	49Y	19	77	smoker	25	pulmonary squamous cell carcinoma	Not mentioned	pT2b, pN2(9/38), L1, V0, Pn0, R0, G3,cM0	right lower lobe	no	lobectomy

TTF-1/p63/p40 expression in the experiment					
Original tumor biopsy			3D model		
TTF-1	p40	p63	TTF-1	p40	p63
−			−		
	+			−	−
Failed, not detected			Failed, not detected		
−			−		
Not detected			Not detected		
	+			−	−
		+		−	+
	+			−	−
+			−		
+			−		
	+			+	
−			−		
+			−		
−			−		
+			−		
+			+/−		
+			+/−		
+			−		
+			+		
−			−		
	−	−		+	−

+	Positive expression
−	Negative expression
+/−	Weak positive
